# The Physical, Chemical and Physiological Limits of Life

**DOI:** 10.3390/life5031472

**Published:** 2015-07-17

**Authors:** Dirk Schulze-Makuch, Alexander Schulze-Makuch, Joop M. Houtkooper

**Affiliations:** 1 School of the Environment, Washington State University, Pullman, WA 99163, USA; 2 Center for Astronomy and Astrophysics, Technical University Berlin, Berlin 10623, Germany; E-Mail: joophoutkooper@gmail.com; 3 Department of Physics, University of Leipzig, Linnéstraße 5, Leipzig 04103, Germany; E-Mail: aschulzemakuch@gmail.com

**Keywords:** physiology, extreme environments, adaptation, Mars, Titan

## Abstract

Life on Earth displays an incredible diversity in form and function, which allows it to survive not only physical extremes, but also periods of time when it is exposed to non-habitable conditions. Extreme physiological adaptations to bridge non-habitable conditions include various dormant states, such as spores or tuns. Here, we advance the hypothesis that if the environmental conditions are different on some other planetary body, a deviating biochemistry would evolve with types of adaptations that would manifest themselves with different physical and chemical limits of life. In this paper, we discuss two specific examples: putative life on a Mars-type planet with a hydrogen peroxide-water solvent and putative life on a Titan-type planetary body with liquid hydrocarbons as a solvent. Both examples would have the result of extending the habitable envelope of life in the universe.

## 1. Introduction

Life on Earth has proliferated into nearly every niche on our planet. However, it does not grow under all conditions near Earth’s surface, and one challenge is to find under which conditions it can actually grow and colonize a habitat and under which conditions it simply can survive waiting until better environmental conditions arrive again. Certainly, life on Earth is very adaptable, which has led to an immense biomass and an incredible biodiversity. Therefore, it is a challenge to find where the limits to this adaptability are.

This paper is a broad and speculative overview of this issue, including a review of two speculations about how radically different life might possibly be, to thrive in environments where life from Earth is not expected to survive. We provide some physiological examples of how the limits of life might be extended and then speculate first about heterotrophs on a dry planet like Mars, where life has adapted to a cold and dry environment by employing hydrogen peroxide as an antifreeze, and secondly speculate about how life might interact with a Titan-like environment, given the physicochemical conditions on the surface of Titan. These speculations serve to illustrate what would otherwise be abstract reasoning; in the case of Mars extending the biochemistry as known from Earth to a more challenging environment on a terrestrial planet and in the case of Titan to propose a novel biochemistry in order to deal with a radically different planetary body. We hypothesize that in the latter case, a different biochemistry would arise with different physical and chemical limits of life. If the hypothesis is correct, this means that life may be found under circumstances where no Terran life could live or even survive. 

When considering the limits of life, we tend to think of the conditions in which individual organisms are able to survive. These limits are relatively easy to observe and to experiment upon. However, we may have to distinguish between the conditions for survival and the more limited conditions under which growth still occurs. The limits of conditions for growth are closely related, but not identical to the limits of organisms being able to complete a life cycle [1]. These limits are even more complex when considering conditions that vary, e.g., due to diurnal and seasonal cycles. Moreover, individual organisms may migrate to seek the conditions suitable for the phase of their life cycle. Life on Earth reveals a great diversity of examples of such survival strategies, which complicates the determination of limits. The survival of individual organisms is often best achieved through association of individuals in groups of the same species and through mutual beneficial relationships between different species, leading to their interdependencies within an ecology. On a larger timescale, it appears that the interplay between the lithosphere, atmosphere, hydrosphere and biosphere leads to a stabilization of the conditions on the Earth’s surface through the biogeochemical cycle, which is beneficial for the survival of existing life.

Broadening the search for extraterrestrial life raises the questions of whether the diversity as observed on Earth reflects the limit of life or whether life elsewhere in the universe could manifest in an even greater diversity [2]. Here, we briefly review what are some of the physical, chemical and physiological possibilities of life on Earth and then speculate on how this pertains to the likely broader range of possibilities in the universe. Finally, we sketch some hypothetical organisms that may even exist in our planetary neighborhood. 

## 2. The Physical and Chemical Limits of Life

On Earth, life is based on carbon as a major building block, water as a solvent and chemical bonds and light as life-sustaining energy sources, which seems to be an ideal combination on a terrestrial planet like Earth, which has an average surface temperature of about 15 °C under a pressure of 1 bar. In principle, other building blocks than carbon are feasible, such as silicon, but these would require very different planetary conditions to be used in a comparable fashion to carbon on Earth. Water as a solvent has great benefits and also some challenges (reviewed by Schulze-Makuch and Irwin [3]), but being the most abundant molecule on our planet that exists in liquid form, life had simply to adapt to some of its drawbacks. Light is plentiful on our planet, and organic compounds with covalent bonds are versatile at average Earth temperatures, thus providing a powerful combination that resulted on Earth in a biosphere with a large biomass and an incredible biodiversity, including complex life. 

There are limits to the conditions under which life can exist on our planet. Most pronounced is the temperature envelope under which active life can exist. Organismic growth can usually occur at temperatures from at least −15 °C to about 113 °C. There are also reports in the literature that the temperature range may even be broader. For example, metabolic activity was inferred down to temperatures of −40 °C due to anomalous concentrations of gases [4], and the upper temperature limit may be as high as about 122 °C. At that temperature, a methanogenic archaea could be cultured under a pressure of 20 MPa [5], only limited by the solubility of lipids in water and protein stability [6]. In principle, if the biochemistry of organisms could be adapted to these extremes, perhaps even higher temperatures may be tolerated, but the practical limit due to energetic and biochemical constraints under which life can still metabolize and reproduce is surely much lower [2]. Hyperthermophilic microorganisms require specialized cell components, like proteins and membranes, to be stable and function at high temperatures. Particularly, at temperatures of 100 °C and beyond, some low molecular weight compounds, such as ATP and NAD, hydrolyze quite rapidly, and thermolabile amino acids, like cysteine and glutamic acid, are decomposing [7]. The pressure tolerance of life, though, is high and extends to at least 1100 bar [8]. 

Organisms, particularly microbes and other microscopic organisms, are quite tolerant to extreme pH values, from just below 0 to about 13. *Ferroplasma* sp. and *Cephalosporium* are examples of organisms that live at low pH-values, *Natronobacterium*, and several species of protists and rotifers are examples of organisms that live at very high pH-values [3,9]. 

However, life on Earth is relatively sensitive to a lack of water: bacteria, archaea and fungi can only metabolize at water activities down to about 0.6 [10]. Adaptation to water with high salt content, however, is quite common, as some Halobacteria and archaea can grow in 35% NaCl solution [3]. 

Another physical limit to life is radiation, both UV and ionizing radiation. Tolerances to radiation vary widely. Tardigrades, microscopic animals that usually live in mosses and lichen, can withstand ionizing radiation doses up to 5000 Gy when in the dormant state [11] and display additional special adaptation traits, such as anhydrobiosis and cryptobiosis [12] (elaborated on below). *Deinococcus radiodurans* can still tolerate higher radiation doses and grow at doses upward of 10,000 Gy. 

The physical and chemical limits of life, as known from Earth, are intrinsically related to its biochemistry, particularly to the physical and chemical properties of water. Thus, if life could exist based on a very different type of biochemistry, for example as proposed for Titan with liquid hydrocarbons as a possible solvent for life [13], then the limits of life in the universe would be much broader.

## 3. Examples of Physiological Limits of Life

Based on the high biodiversity shown by life on Earth, in the past and at present, one might ask what the physiological limits of life are. This is very difficult to elucidate, because these limits depend largely not only on the environment, but the whole ecosystem, including other organisms and the exchange of metabolites between them. Thus, we will not attempt here to generally characterize what the physiological limits of life on Earth are, but will instead give two examples that show that these limits can be much broader than generally known. The two examples given are relatively complex multicellular life forms; single-cellular life forms are generally known to be able to adapt to even more extreme conditions.

### 3.1. Survival under Extreme Conditions in a Dormant State

Several animals can enter an ametabolic state called anhydrobiosis, which allows them to survive a lack of water for long periods of time. For example, recovery from anhydrobiosis was reported from tardigrades and rotifers after nine years, crustaceans after 16 years, insects after 17 years and a nematode after 39 years [14,15,16,17]. Tardigrades are a good example of the use of the anhydrobiotic state. This state is usually initiated by a lack of water. The body water content of tardigrades can decrease to between one and three weight percent [18] with the contracted animal, or “tun”, not showing any visible signs of life, yet when exposed to a drop of water, activity is resumed [19]. Anhydrobiotic measures that are invoked in the case of water loss usually include the use of low-molecular mass compatible solutes, including trehalose [20]. Trehalose has a high solubility, a low reactivity and a low tendency to crystallize, substituting for bound and free water and, thus, maintaining the structures of cell membranes and proteins. Disaccharide trehalose has been measured at concentrations of up to 10 to 20 weight percent in several animals that can initiate an anhydrobiotic dormant state, including nematodes [21], insect larvae [22] and embryos of the brine shrimp *Artemia salina* [23]. However, only a relatively small amount of trehalose is found in tardigrades [24]. It appears that tardigrades rather utilize heat shock proteins and late embryogenesis abundant (LEA) proteins, which can serve as molecular chaperones and help protect proteins from denaturation [11,25,26].

Although the invoked biochemistry is not revealed yet to a satisfying level on how these small animals survive the lack of water, the environmental stresses that can be survived are simply astounding: tardigrades can survive extremely high temperatures of up to 151 °C [18], but also very low temperatures, down to nearly absolute zero [27]. They survive pressures of up to 6000 bar [28], but also vacuum conditions. Jönsson *et al.* [29] reported that tardigrades can survive the vacuum of open space plus space radiation of low-Earth orbit for at least 10 days. The survival of the dormant state may also be much longer than nine years. Franceschi [30] reported the recovery of tardigrades from a dry moss sample taken from an Italian museum after 120 years of preservation, though the individual organisms underwent “quivers” in several zones of their body. 

### 3.2. Hydrozoans and Their Induction of Reverse Development

A well-known phenomenon in animal development is metamorphosis, a biological process by which an animal physically develops a conspicuous and relatively abrupt change in its body structure through cell growth and differentiation. Organisms that undergo metamorphosis, which is usually accompanied by a change of habitat or behavior, include some insects, amphibians, mollusks, crustaceans, cnidarians, echinoderms and tunicates. Metamorphosis, for example in a butterfly, would be physiologically remarkable enough for anyone who has not encountered this before. The downstream development of an organism from zygote to adult is in most species irreversible. 

However, even physiologically more remarkable is that cnidarians have the exceptional capability for reverse development, that is to briefly shift their downstream development to the reverse ontogenetic direction. Specifically, by means of morphological reorganization, the current life stage of the cnidarian is transformed to its preceding life stage. Such a transformation is triggered by unfavorable conditions in its environment. Although, most species that exhibit reverse development are bound by restriction, *i.e.*, when and to what extent reverse development can occur. One particular hydrozoan, *Turritopsis dohrnii*, can undergo complete reverse development. 

The normal life cycle of *Turritopsis dohrnii* is as follows [31]:

(1) Polyp colonies bud gonochoric medusa; in time, these are released into the free water column.
(2) The mature medusa release their gametes and then usually die.
(3) After fertilization, the gametes transform to planula larva.
(4) Through metamorphosis, triggered by an external bacterial signal, a mature planula larva transforms to a primary polyp.
(5) The primary polyp then becomes a new polyp colony.

If the medusa is affected by environmental stresses, such as a change in salinity, change in temperature, starvation, mechanical shock, epigenetic induction or endogenous induction that is activated upon reaching sexual maturity, then the medusa will transform back to a polyp to have a better chance of survival than the free-floating medusa. The polyp will then once again follow the normal life cycle. It is intriguing that the polyp colony and all medusa derived from it will be genetically identical to the medusa that back transformed to the primary polyp.

The phenomenon of metamorphosis is seen in many species, and indeed, the reverse development in *Turritopsis dohrnii* has parallels to a reverse metamorphosis. The parallels include the utilization of apoptosis in both metamorphosis and reverse development, the presence of artificial inducers, e.g., cesium ions and heat shock, in reverse development and larvae metamorphosis. Furthermore, the ability of the cells to *trans*-differentiate is highly important in both processes. 

As pointed out above, a complete life cycle should be considered when determining the limits of life for an organism [1]. This is not the case in the above example. Nevertheless, it is an incredible example of physiological adaptation by reversing the life cycle to overcome environmental stress. Furthermore, if the medusa state of the life cycle were optional and reproduction could also occur in the polyp state, then the argument could be made that a life cycle is completed, and the environmental conditions would fall within the limits of that organism. While this is not the case for *Turritopsis dohrnii*, it could be the case for other organisms that undergo reverse metamorphosis. Metamorphosis and reverse metamorphosis, apparently involving multiple body plans, may be more generally usable strategies to adapt to changes in the environment and the need of organisms to grow and reproduce.

## 4. Examples of Hypothetical Organisms

Here, we hypothesize on organisms that may exist on other planetary bodies, but we will limit ourselves to two examples, hypothetical organisms using a hydrogen peroxide-water solvent on a cold and dry Mars-like world and organisms that may live in liquid hydrocarbons on a Titan-like world. Other examples of hypothetical extraterrestrial organisms, such as floaters in the atmosphere of a gas giant [32], possibilities of life in supercritical carbon dioxide as a solvent for life [33], or in fluorine-rich environments [34], or within the ocean of an ice-covered moon, can be found in the literature [3,35,36]. As life and its environments are intrinsically interwoven, we base the hypothesized organisms on environments of nearby planets and moons to provide some scientific basis for our speculation. We do not propose that these organisms exist, but like to point out that their existence would be consistent with physical and chemical laws, as well as biology. Our given examples for Mars and Titan, respectively, are testable with future space missions, some of which are in development, like ExoMars.

### 4.1. Lithotrophic and Heterotrophic Life on a Planet like Mars

Mars is a terrestrial planet with many similarities to Earth, especially early in its natural history, when it was warmer and wetter [37]. Today, the near-surface environments of Mars are similar to some of the driest desert environments on Earth, such as the Atacama Desert and the Dry Valleys of Antarctica, but Mars is even more dry and challenging. Nevertheless, life on Earth with its biochemical toolset might still be able to handle Martian environmental conditions, perhaps with a few novel adaptations not seen on Earth.

One option to cope with these more challenging environmental conditions would be to invent new types of adaptation. Based on the Viking Lander Biology Experiments [38], Houtkooper and Schulze-Makuch [39] previously hypothesized that organisms might utilize a water-hydrogen peroxide (H_2_O-H_2_O_2_) mixture rather than water as an intracellular liquid in the current Martian environment. Using a water-hydrogen peroxide mixture would have the particular advantages in this cold and dry environment of providing a low freezing point, a source of oxygen, energy and hygroscopicity. Hygroscopicity would be especially advantageous, because it could provide organisms the ability to attract water directly from the atmosphere, analogous to how microorganisms in the Atacama Desert use hygroscopic salt crystals [40]. An adaptation to highly oxidative compounds would also give microorganisms living near the surface of Mars an adaptive edge to counter radiation damage, as the biochemical protection against oxidants and radiation (which produces oxidants) is complementary. Hydrogen peroxide has been measured in the Martian atmosphere [41], but is metastable. Nevertheless, hydrogen peroxide concentrations are thought to be as high as 25–250 ppm, either adsorbed or chemically bound at the ground surface [42].

In the Martian-type environment, photoautotrophic microorganisms may exist, perhaps using a similar life-style to the endoliths in Antarctica, which produce their biomass from the CO_2_ and H_2_O in the atmosphere and some photochemically-produced nitrate in the soil. The biomass would mostly consist of the stabilized proteome and DNA in the H_2_O_2_-H_2_O mixture of their intracellular fluid. The hydrogen peroxide is thought to be produced by photosynthesis involving the possible reactions:

CO_2_ + H_2_O → CO + H_2_O_2_ (1)

CO_2_ + 6 H_2_O → CH_4_ + 4 H_2_O_2_ (2)

CO_2_ + 3 H_2_O → CH_2_O + 2 H_2_O_2_ (3)

Equation (2) would be most efficient to produce the liquid solvent H_2_O_2_, which is limited on Mars, while Equation (3) may produce formaldehyde or, with the same overall composition, carbohydrates, or saccharides. As this is a metabolite from the hydrogen peroxide production, it might be put to good use by organisms having cell walls consisting mainly of polysaccharides within an excreted mass of extracellular polysaccharides (EPS). During the daytime, these organisms may photosynthesize H_2_O_2_, with CH_4_ as a metabolite, which is consistent with the recent observation that higher spikes of methane were observed during day-time measurements by the Curiosity Rover than during the nighttime [43]. As the relative humidity is high during the nighttime, the CH_4_ is scavenged from the atmosphere to build more EPS (EPS being produced by the overall equation: CH_4_ + CO_2_ → 2 CH_2_O, ΔG = −493 kJ/mol), while a putative water-absorbing perchlorate-peroxide cycle also sets in. 

While the perchlorate found in deserts on Earth may be produced by atmospheric chemistry, the mechanism for its formation on Mars is still unclear [44]. Whereas on Earth, perchlorate metabolism is well known, the reverse process of microorganisms producing perchlorate is not. In principle, the perchlorate ion can decompose exothermically into dioxygen and chloride, but the activation energy for this reaction is so high that under normal circumstances, the perchlorate ion is very stable. For Mars, the presence of perchlorate is a greater puzzle, because of the paucity of ozone and water vapor in the Martian atmosphere. Therefore, it is not far-fetched to hypothesize that hydrogen peroxide-containing organisms on Mars might employ a cycle via Reaction (4), which is, in principle, reversible, to produce the hygroscopic extracellular perchlorate to help scavenge water from the atmosphere, while the H_2_O is kept within the cell.

4 H_2_O_2_ + Cl^−^ ↔ 4 H_2_O + ClO_4_^−^, ΔG = −362 kJ/mol (4)

The reverse (endothermic) reaction can be used when a source of energy, e.g., sunlight, is available to produce the intracellular H_2_O_2_ as an antifreeze and available store of energy. Alternatively, perchlorate could be retained inside the cell to avoid dehydration and to take over some of the proposed functions of H_2_O_2_, as described above. On Mars, the advantage of producing perchlorate is that it is hygroscopic and helps attract water from the atmosphere, since the day-night cycle involves a period with low temperatures and a high relative humidity [45]. 

On Earth, it is often observed that under harsh conditions, symbiosis is more likely to occur than predator-prey relationships, but perhaps the following speculation of a heterotrophic small animal, let us call it a bombardier beetle-like organism, is an exception (and very unlikely on Mars today, due to the general lack of potential nutrients and the high radiation environment). While the motile organism may crawl or walk over small distances, when a local source of food is exhausted, it might sense the decomposition products of perchlorate (oxychlorines) and jump in the direction of the perchlorate-rich patches, where the perchlorate-hydrogen peroxide-cycling organisms are to be found. Since the sources of nutrients are few and far apart, the organism must be mobile over macroscopic distances and have thus invented a suitable mechanism of locomotion, in order to survive and reproduce. Clearly, the consumption of their prey is not easy, as reducing and oxidizing components would have to be kept apart. Therefore, they might first suck out the liquid cell components, filter the H_2_O_2_-rich mixture and store this in a chitinous chamber, with some analogy to the bombardier beetle on Earth. In a second stage, the EPS and other solid components of their prey may be dissolved and stored in liquid form.

For their jumps, the bombardier-beetle-like organism could possibly use rocket propulsion. On Earth, bombardier beetles store an aqueous solution of hydroquinone at a 25% mass concentration and hydrogen peroxide at a 10% mass concentration in a reservoir. The solution can be squeezed into a reaction chamber and mixed with enzyme catalysts (catalase and peroxidases), resulting in a very fast catalytic reaction that heats the solution to the boiling point, where then it is discharged for defensive purposes [46]. This reaction cannot be used by the bombardier beetle for propulsion, because it does not provide enough thrust. However, on another planet or moon, this principle could be modified and applied to allow the organism to lift off, especially on planets and moons with lower gravity. 

For example, squids have been documented to launch themselves from the water using rocket propulsion. The squid intakes a large volume of water in its mantle and then by muscular contraction forcibly expels the water through a funnel that vectors the water jet [47]. The force of propulsion is F = Ju, where u is the average velocity of the expelled mass and J is the mass flow rate. J is equal to ρuA, where ρ is the density of the mass and A is the area of the funnel [48]. This mode of locomotion seems more favored in environments of dense fluids, unless an alternative method for accelerating the expelled mass to a higher velocity is used. Chemical reactions are thus an option, but this approach is likely not used by the bombardier beetle, because of the difficulty to control chemical reactions and because a large amount of energy would be needed to produce the reactants. However, if a hypothetical organism can acquire the reactants directly from the environment and has to overcome larger distances, then there is no reason why chemical rocket propulsion is not a valid mode of locomotion. 

In such a suitable planetary environment, the reaction chamber may be lined with EPS for protection and cooling of the chamber wall and for providing extra reaction mass. The expelled reaction mass may reach an exhaust velocity of well over 1 km/s. With a powerful jump, as much as 5% of the organism’s mass could be expended. This could well result in an attained velocity of about 50 m/s. Without just minimal friction from a thin Martian-like atmosphere, the maximum attainable height by a vertical jump under the Martian surface gravity conditions would be about 300 m, allowing them to cover hundreds of meters by a single reaction. 

### 4.2. Large Single-Cell Organisms on a Moon like Titan?

On a moon like Titan, with its surface being partly covered by liquid methane and ethane, organisms with a biochemistry as known from Earth would not be expected to thrive, even if some novel adaptations, as speculated in the previous example, were utilized. Certainly, a non-polar solvent would require that the biochemistry of these putative organisms be drastically different, and a new biochemistry would have to be invented. Organisms on Earth have membranes that are amphiphilic with their polar (hydrophilic) heads immersed in the solvent water and their non-polar (hydrophobic) tails oriented toward each other, away from the solvent. The membranes have the important biological function to take up nutrients, respond to intracellular signals and discard wastes. If a non-polar solvent is used by life, the chemical orientation might be reversed, analogous to reverse micelles, or may consist of multi-layered lipids [49]. These membranes may incorporate silanes as building blocks at the environmental conditions existing on Titan. Processes that could create silanes under the extremely cold and reducing conditions on Titan include serpentinization reactions in a cryogenic environment, meteorite impacts, ice-silicate grains exposed to UV irradiation and 1 MeV protons in a hydrogen atmosphere [50]. Furthermore, Stevenson *et al.* [51] recently suggested membranes composed of small organic nitrogen compounds, azotosomes that would be capable of forming and functioning in liquid methane at cryogenic temperatures. 

Metabolic reactions would likely have to yield more energy in such a cold environment to yield sufficiently high reaction rates, and catalysts would play a significant role to overcome the activation energies. Schulze-Makuch and Grinspoon [52] suggested the catalytic hydrogenation of photochemically-produced acetylene as one possible metabolic pathway on Titan. Acetylene is a compound not often used for experiments at room temperatures, because it is highly energetic (explosive). However, under extreme cold, it could provide sufficient energy to sustain an organism. Acetylene is produced high in Titan’s stratosphere from solar UV radiation and, then, for the most part, condenses and falls to the surface, thus providing potential means of transferring high-altitude solar UV energy to surface chemical reactions [49]. Alternatively, radical reactions may be utilized as possible metabolic pathways on Titan [52]. Raulin [53] suggested that Titan’s atmosphere is an active site of complex carbon and nitrogen radical chemistry. On Earth, radical reactions are rarely used as part of a metabolic pathway, but on an extremely cold world, they might just be what is needed to get metabolism going. Possible radical reactions would be:

CH_2_ radical + N_2_ → CN_2_H_2_ (5)

and:

2CH radical + N_2_ → 2HCN (6)

which would have the further advantage to produce the two biologically-important compounds cyanamide and hydrocyanic acid, respectively. Various pathways have been proposed for assembling amino acids and proteins or their ammono-analogues using cyanamide and HCN as basic compounds [54].

The availability of hydrocarbons as a solvent of life might improve the chances for the origin of life, because the assemblage of organic macromolecules that could give rise to life appears to be more straightforward in a hydrocarbon environment [3]. Organic synthesis reactions would occur much more frequently and proceed to a higher complexity than in a water environment. The lack of oxygen in a Titan environment may not necessarily be a disadvantage, because if unbound oxygen has an extremely low availability, oxygen-containing molecules might be replaced with nitrogen analogues in a reducing Titan environment [55,56]. Further, the microscopically small cell as a basic unit of life on Earth may be a misleading model for life in some non-aqueous environments. In an extremely cold, hydrophobic (but liquid) environment, surface-to-volume ratio considerations may be less constraining than at higher temperatures in polar solvents. On Earth, when the solvent becomes more viscous, at lower temperatures, diffusion slows down together with the rate of metabolism [40]. If this also applies to Titan, life on a Titan-like world could involve huge (by Earth standards) and very slowly metabolizing cells [57]. Furthermore, there is no pre-defined age limit in biology. Evolution for organisms on a Titan-type world with a very slow metabolism and an environment where not much radiation damage is occurring may occur much slower, possibly raising the life span of individual organism on that moon significantly. 

## 5. Discussion and Conclusions

We are far away from having mapped the biological inventory on our home world. New organisms are still being discovered on a regular basis today, especially in environments that are inhospitable for us and that are difficult to access, such as the deep sea environments. The last hundreds of millions of years of evolution on Earth provided us with a rich biodiversity of organisms, which explored a huge set of biochemical possibilities. Yet, our biosphere and the adaptations we observe are probably only a small subset of what is possible in biology. Life is intrinsically interwoven with its environment, so we can assume that during billions of years of natural history, many or nearly all of the biochemical possibilities were explored that are possible on a terrestrial planet with an average surface temperature of 15 °C, 1 bar pressure and an oxygenated atmosphere (in the later part of Earth’s natural history). Many Terran organisms live at environmental conditions far removed from this average, but these are likely only adaptations of life’s general biochemistry on Earth to extreme environments, which essentially means a deviation from the “average” conditions on our planet. The observation that these extreme environments were populated shows the flexibility of Earth’s biochemistry and the power of evolution. Nevertheless, our biochemistry has limitations, as observed in extreme environments and inhospitable conditions, but some organisms are very resourceful in overcoming these challenging environments by processes, such as reverting into a dormant ametabolic state or reverse metamorphosis. How far can a biochemistry be stretched? To make a living on today’s cold and dry Mars, bathed in radiation, would some novel adaptations do the trick, or would, like in the case of Titan, a much different biochemistry be needed, because the environmental conditions are just too different?

One point arguing for the flexibility of organisms or the “stretchability” of a biochemistry is that organisms can become dormant if the environment temporarily does not fit their needs; or in the case of animals, which solved the problem of uninhabitable conditions by moving from one habitable location to another, bypassing uninhabitable locations for them on the way. Microorganisms that do not have a means of self-propelled movement use natural means, such as flowing water or wind, as a way to move to other habitable locations (and if stranded in an uninhabitable location, often use the strategy to become dormant until either they are transported back into a habitable location or environmental conditions change in a way that their present location becomes habitable for them again). Thus, an environment does not need to be continuously habitable to be inhabited by organisms, taking both variations of time and locality, in regard to habitability, into account. 

The diversity of organisms present within a biosphere can be manifested in many ways, for example biochemically, morphologically and behaviorally (lifestyle). This would also be expected to be the case on an exoplanet with the result that it would broaden the planetary environments that can be used as habitats. Additionally, new environments are likely to be opened for habitation, some of which would be considered uninhabitable by Earth standards. 

The question arises that, if the average environmental conditions would be different on some other planet, would a different biochemistry evolve with different physical and chemical limits? Other planets and moons would be a testing ground for this hypothesis. For example, if life exists on Mars or Titan, how different would it be? The testing of the hypothesis would be challenging for Mars, because about 4.3 billion years ago, when life may have originated there, environmental conditions were quite similar to Earth’s [58]. During the last billions of years, environmental conditions changed drastically to the cold and dry planet we observe today [37]. A further complication is the possibility of microbes being transported by panspermia between the two planets [3,59], thus if life exists on Mars today, it may be related to Earth life [60]. Either way, life may have existed on Mars long ago with a very similar or the same biochemistry as on Earth and only evolved more recently adapting to the current conditions. The hypothesized microorganisms based on a hydrogen-peroxide-water mixture as a solvent detailed above would be an example of such a later adaptation; or the temporarily and locally non-habitable conditions on Mars (at least as far as we understand them) could be bridged through a combination of dormancy and physiological adaptation.

The hypothesis of how large the landscape of life may be could better be tested with life that might exist on Titan. The environment is drastically different from Earth, being an extremely cold, organic-rich and reducing environment. Possible solvents only include the hydrocarbon compounds on the surface, mostly in the form of lakes and likely ammonia-water slurries in the subsurface. If life can exist there, which might be expected based on the available chemistry [8], but perhaps limited by the poor availability of metal catalysts [61], it would have to have a different type of biochemistry. Example possibilities are discussed in Section 4.2. The properties of the solvent will be the major constraint on the physico-chemical limits of life. In general, if life can originate and persist under such environmental conditions, vastly different from those exhibited on Earth, then only a small subset of the possibilities of life are plotted out on Earth. The landscape of physiological possibilities is much more difficult to assess. On Earth, we still do not know of all of the physiological options various organisms have, but the fraction of what is known is astounding. Two amazing, but manifested examples in Earth’s biology are given in Section 3. We chose complex life forms as examples, because these underline the point that even complex organisms, such as tardigrades, can live in environments that are uninhabitable for long periods of time. Microbes are known to live in even more extreme conditions. What would be the limit of their physiology? How would these physiological adaptations increase the number of environments that would not be habitable without them? This is difficult to assess, but an adaptation, such as anhydrobiosis or cryobiosis, would clearly be a critical benefit if the planetary body undergoes elongated seasons of extreme dryness or low temperatures, respectively. The physiological possibilities are likely a function of the biodiversity experienced on a planet and probably of the length of time evolution was able to work on these types of solutions to environmental challenges. If a planet has only a biosphere restricted to certain niche habitats, such as perhaps on Mars, fewer physiological adaptations may be expected. On a planet with a rich global biosphere, many of these physiological adaptations, as invented on Earth, may exist there as well, as these adaptations may be rather independent of which information code for replication is used.

Only the discovery of extraterrestrial life and a second biosphere will allow us to test these hypotheses, which would be one of the grandest achievements of our species.
